# Anesthesia for Endovascular Therapy for Stroke

**DOI:** 10.3390/neurolint16030050

**Published:** 2024-06-20

**Authors:** Arianna Gaspari, Giulia Vaccari, Federica Arturi, Gabriele Melegari, Stefano Baroni

**Affiliations:** 1 Department of Anesthesia and Intensive Care, Azienda Ospedaliero Universitaria di Modena, Via del Pozzo 71, 41125 Modena, Italy; gaspari.arianna@aou.mo.it (A.G.); s.baroni@libero.it (S.B.); 2 School of Anesthesia and Intensive Care, University of Modena and Reggio Emilia, Via del Pozzo 71, 41125 Modena, Italy; vaccarigiulia9@gmail.com (G.V.); federica.arturi.94@gmail.com (F.A.)

**Keywords:** stroke, conscious sedation, endovascular therapy

## Abstract

Background: In patients with acute ischemic stroke, the standard of care is to perform intra-arterial endovascular thrombectomy in addition to intravenous thrombolysis. In this study, we investigated the different anesthetic techniques chosen for this procedure and clinical outcomes. Methods: Patients undergoing endovascular procedures were divided into three groups. The first group consisted of patients who received general anesthesia, the second group underwent the procedure under conscious sedation and local anesthesia at the catheter insertion site, and lastly the third group included patients who received only local anesthesia at the catheter insertion site, without sedation. Results: During the endovascular procedure, we did not notice significant differences in vital parameters, in particular the mean blood pressure (MAP) between patients treated with different types of anesthesia. Also, the duration of the revascularization did not show significant differences between the three groups. The main point is the absence of differences in terms of functional and clinical outcomes, using various scores as reference, such as the National Institutes of Health Stroke Scale (NIHSS) score at 7 days, NIHSS and Modified Rankin Scale (MRS) at time of discharge, and MRS after 3 months. These scores did not show significant differences in groups treated with different types of anesthesia. Conclusions: The rate of success of the revascularization procedure is almost overlapping between patients treated with conscious sedation and general anesthesia. In addition, we did not notice significant differences between groups in terms of functional and clinical outcomes. Considering the possible usefulness of applying conscious sedation, at OCSAE of Baggiovara, an internal protocol for conscious sedation was introduced to standardize the treatment in patients undergoing endovascular procedures.

## 1. Introduction

Stroke is the second cause of death and the first cause of disability in Europe. The incidence of acute ischemic stroke is increasing due to aging of the population. The prevalence of stroke in Europe adjusted for sex was estimated at 9.2% (95%CI: 4.4–14.0), respectively, 9.1% (95%CI: 4.7–13.6) in men and 9.2% (95%CI: 4.1–14.4) in women [1].

In patients with acute ischemic stroke and large vessel occlusion, the standard of care is to perform intra-arterial endovascular thrombectomy in addition to intravenous thrombolysis, if possible [2,3].

During endovascular treatment, it is important to keep the patient as still using a particular type of anesthesia or sedation. The available options include general anesthesia (GA), conscious sedation (CS) with spontaneous breathing, or local anesthesia for vascular access, with or without sedation. Whether the anesthetic strategy during endovascular therapy has an impact on the outcome of the patient is a matter of discussion. General anesthesia provides control over the immobility, airway, oxygenation and ventilation, and ability to fully control procedural pain. However, it could lead to hemodynamic instability, respiratory complications, and delayed endovascular treatment [4,5,6]. On the other hand, conscious sedation allows for real-time neurologic examination, shorter time to treatment initiation, and more hemodynamic stability.

Several studies have compared general anesthesia with conscious sedation and had controversial results. As such, both anesthetic strategies could be used, based on the patient’s clinical features, hospital protocols, and physician assessment, to achieve the best possible outcome [7,8]. 

At our center, we realized that different physicians used a variety of anesthetic drugs for conscious sedation. We developed an internal protocol in January 2019 to ensure consistency and safety. The protocol is based on propofol and remifentanil, and is described in Appendix A.

In this retrospective observational monocentric study, we described and investigated the anesthetic technique chosen by anesthesiologists for endovascular therapy as well as clinical features and clinical outcomes of patients who underwent this procedure from 1 January 2019 to 31 May 2019, in Azienda Ospedaliero Universitaria OCSAE of Baggiovara (MO).

### 1.1. Outcomes

#### Primary Outcome

This study describes an overview of the characteristics of patients with ischemic stroke who underwent endovascular therapy under a conscious sedation regime. This study recorded sex, age, comorbidities of patients, stroke severity, extension, and location. The patient’s vital signs were monitored throughout their admission, the procedure, and the 24 h post-procedure. The primary objective of this study was to observe whether there are any differences between the possible anesthetic strategies in terms of the clinical outcome and duration of the revascularization procedure. This study aims to investigate the possible existence of differences between groups in terms of perfusion indexes, especially the lactates, mean arterial pressure (MAP), Glasgow Coma Scale (GCS) score, National Institutes of Health Stroke Scale (NIHSS) score on admission and discharge, and Modified Rankin Scale (MRS) score.

## 2. Materials and Methods

### 2.1. Patients

We analyzed patients aged 18 or older who provided written informed consent and were admitted to our center for endovascular therapy for ischemic stroke from 1 January 2019 to 31 May 2019. 

We included patients who underwent the procedure under general anesthesia, conscious sedation, or local anesthesia. 

### 2.2. Protocol for General Anesthesia

All patients were treated with propofol in a bolus, Fentanyl and Rocuronium for anesthesia induction, and propofol and remifentanil in a continuous infusion as maintenance. The depth of sedation was monitored with a bispectral index (BIS^©^) reading [7], which was kept between 40 and 60.

### 2.3. Protocol for Conscious Sedation 

The protocol for conscious sedation utilized by our anesthesiology team involves local anesthesia with 2% lidocaine at the catheter insertion site and a continuous infusion of propofol 1%, with a top-up bolus administered as needed. Additionally, remifentanil continuous infusion can be used if necessary. 

Patients were kept comfortable and quickly awakened by achieving a Richmond Agitation–Sedation Scale (RASS) score between 0 and −2. The depth of sedation was also monitored with a bispectral index (BIS^©^) reading [7], which was kept between 60 and 80.

### 2.4. Protocol for Local Anesthesia

For this group of patients, anesthesia included a local application of lidocaine 2%, performed directly by the neuroradiologist.

### 2.5. Study Design 

Patients with acute ischemic stroke undergoing endovascular procedures were divided into three groups based on anesthetic techniques. 

The first group consisted of patients who received general anesthesia. This group included patients with a Glasgow Coma Scale score less than 8 upon admission, those with posterior circulation strokes, and those who presented with psychomotor agitation, convulsions, or vomiting and were already intubated by the emergency team. This group also included patients for whom conscious sedation or local anesthesia was not possible.

The second group of patients underwent the endovascular procedure under conscious sedation and local anesthesia at the catheter insertion site. To maintain patient safety, our internal protocol was followed, and additional measures were taken based on current international guidelines. 

The third group included patients who received only local anesthesia at the catheter insertion site, without sedation, administered by the neuroradiologist [9]. 

After the procedure, patients with an Aldrete score greater than 8 or those who received general anesthesia were admitted to the ICU.

During this study, we collected epidemiological characteristics of the population, including age, sex, comorbidities, the Glasgow Coma Scale (GCS), the degree of clinical instability of the patient at admission according to the Modified Early Warning Score (MEWS), the risk of mortality in critically ill patients on admission according to the Sequential Organ Failure Assessment Score (SOFA SCORE), the location of the ischemic stroke, the severity of the neurological deficit of the ischemic stroke measured by the value on admission on the NIH stroke scale (NIHSS), the type of treatment performed (intra-arterial mechanical thrombectomy or both intra-arterial mechanical thrombectomy and intravenous pharmacological thrombolysis), and the degree of disability at admission according to the Modified Rankin Scale (MRS). We evaluated these parameters at admission and after 24 h.

We evaluated revascularization time, sedation protocol adherence, conversion to general anesthesia, post-neurological evaluation time, symptom correction degree, adverse events, complications, and need for ICU hospitalization.

### 2.6. Statistical Analysis

The statistical analysis was performed using SPSS 2.0^©^ software. Descriptive data are presented in tables. The quantitative data were analyzed based on the normal or non-normal characteristics of the collected data, depending on the type of variables observed (categorical or numerical; paired or independent) and the sample size. 

Categorical variables are summarized in descriptive tables showing numbers and percentages. Statistics of continuous variables are expressed as the mean and standard deviation, median, and interquartile range. 

The Student *t*-test or one-way ANOVA test was used to compare normal independent continuous variables, while the Mann–Whitney test or Kruskal–Wallis test was used for non-normal independent continuous variables. Normal paired continuous variables were compared using the paired Student *t*-test and non-normal paired continuous variables were analyzed with the Wilcoxon signed-rank test or Friedman’s ANOVA test. Values with a statistical significance level of *p* value < 0.05 were considered significant.

## 3. Results

This study observed 58 patients, out of which 32 (55.17%) were male, and their average age was 71.22 (±17.11 standard deviation, SD). During the procedure, 8 (13.79%) patients received general anesthesia, 32 (54.2%) were under conscious sedation, and 18 (30.5%) received local anesthesia without sedation. Among patients who received conscious sedation and local anesthesia, five (15.6%) and two (11.1%) patients, respectively, had complications that required conversion to a general anesthesia regimen during the procedure.

Out of all the patients, 53 (91.23%) had cardiovascular comorbidities and 16 (28.07%) had neurological pathologies. Many of the patients had multiple comorbidities. The general impairment indices at admission, such as the MEWS (mean of 1.40 ± 1.5 SD) and SOFA SCORE (mean of 2.26 ± 1.75 SD), did not show significant differences in the subgroups with different intra-procedural anesthetic management. Indices of neurological impairment and agitation on admission, such as GCS (12.30 ± 2.36 SD) (13, 11–14 IQR), NIHSS (14.91 ± 6.74 SD) (14.9 ± 6.7 IQR), MRS (0.21 ± 0.52 SD) (0, 0–0 IQR), and RASS (0.00 ± 0.73 SD) (1, 0–0 IQR), were also similar across the subgroups.

The most involved artery was the middle cerebral artery (36, 62.7%), followed by the obstruction of a branch of the posterior cerebral circulation (7, 12.07%), simultaneous occlusion of the internal carotid artery and the ipsilateral middle cerebral artery (7, 12.07%), isolated lesion of the internal carotid artery (5, 8.62%), simultaneous lesion of the middle cerebral artery and cerebral artery ipsilateral anterior artery (2, 3.45%), and occlusion of an anterior communicating artery (1, 1.72%).

These data are all summarized in Table 1.

During the endovascular procedure, the systolic blood pressure values tended to be lower in all groups compared to admission. However, there was no statistically significant difference in the pre- and post-reperfusion mean arterial pressure (MAP) values (pre-reperfusion: 103.64 ± 12.69 SD in AG, 100.53 ± 11.82 SD in CS, and 104.38 ± 11.89 SD in AL, *p* = 0.411) (post-reperfusion: 86.50 ± 16.58 SD in AG, 90.01 ± 14.70 SD in CS, and 91.06 ± 10.99 SD in AL, *p* = 0.657) among the local anesthesia, conscious sedation, and general anesthesia groups. 

The duration of the revascularization procedure, from the time of vessel puncture to recanalization, in patients submitted to general anesthesia was 144.44 ± 94.97 SD minutes. Although the duration appeared to be longer in patients in the CS group (86.16 ± 59.84 SD), this value was not statistically significant (*p* = 0.072).

At discharge, there was no difference between the groups in terms of functional and clinical outcomes. The National Institutes of Health Stroke Scale (NIHSS) score at 7 days was 7.85 ± 4.59 SD in patients treated with general anesthesia and 11.62 ± 9.05 SD in those treated with conscious sedation, with a difference that is not statistically significant (*p* = 0.374). We observed similar results for NIHSS and Modified Rankin Scale (MRS) at time of discharge and MRS after 3 months: in all instances, the differences between GA and CS groups were not statistically significant. Also, regarding the survival rate, the difference between groups was not statistically significant: 25% in the GA group and 48.08% in the CS group, with a *p* value of 0.824. 

Table 2 includes paramethers at ammission, while Table 3 includes paramethers and neurological outcomes before and after the procedure.

## 4. Discussion

This study is a retrospective observational single-center analysis of 58 patients who underwent revascularization treatment for acute cerebral ischemia, under general anesthesia, conscious sedation, or local anesthesia in an angiography room. The primary objective of this study was to observe whether there are any differences between the possible anesthetic strategies in terms of the clinical outcome and duration of the revascularization procedure. In addition, this study aims to investigate the possible existence of differences between groups in terms of perfusion indexes, especially the lactates, mean arterial pressure (MAP), Glasgow Coma Scale (GCS) score, National Institutes of Health Stroke Scale (NIHSS) score on admission and discharge, and Modified Rankin Scale (MRS) score. Traditionally, patients with alterations in the posterior cerebral circulation have been managed under general anesthesia, while the rest have been treated under local anesthesia or conscious sedation based on the clinical evaluation performed upon arrival in the angiography room. As 61% of the cases involved lesions affecting a middle cerebral artery, the anesthetic management decision was in line with the protocol, with more than half of the patients being treated under conscious sedation. The conversion rate to general anesthesia overlaps with the results of the SIESTA trial [4,10,11]. Various studies in the literature investigate the anesthesiological conduct during the endovascular treatment in the case of ischemic stroke. In particular, three big trials were built to evaluate what might be the best strategy between the two main anesthesiologic conduits adopted in revascularization procedures, general anesthesia and conscious sedation. The AnStroke trial investigated the possible impact of anesthesia on the neurological outcome of these patients, showing no differences between the two groups 3 months after the ischemic stroke [12,13]. On the other hand, the GOLIATH trial seems to observe a worse general outcome in patients treated with general anesthesia. This could be attributed to hemodynamic changes induced by anesthetic drugs, with a consequent prolonged hypotension that may have a long-term impact [14,15]. The SIESTA trial shared the same hypothesis, underlying the superiority of conscious sedation in terms of safety and neurological outcome. In particular, Schönenberger et al. in their trial used the National Institutes of Health Stroke Scale (NIHSS) after 24 h as a predictor of the long-term outcome [5]. Different authors focused on the differences between the two main anesthesiological conducts used during the endovascular treatment. In particular, Campbell et al. showed a superiority of general anesthesia in terms of the success rate of the procedure and recovery of neurological functions, probably due to reduced brain metabolism induced by propofol and sevoflurane, anesthetics used for general anesthesia [16]. This runs counter to the observations of Chabanne et al., which proved an overlap in terms of the survival rate and functional recovery between patients treated with general anesthesia and conscious sedation [17]. Liang et al. focused on different complications related to anesthesia. In particular, they highlighted a higher risk of hypotension and hemodynamic instability in patients treated with general anesthesia. On the other hand, a greater incidence of neurological complications, such as sudden moves or dysphoria, was observed in patients submitted to conscious sedation. No differences were observed between the two groups regarding other complications, such as respiratory infections or hemorrhages [18]. Li et al. in their trial concentrated on the probable variables involved in the mortality rate among patients submitted to an endovascular procedure, identifying general anesthesia as a predictor of increased risk of death. This may be attributed to a longer timeline before the start of recanalization, needed for anesthesia induction and endotracheal intubation, or a possible reduction in blood flow induced by anesthetic drugs, both intravenous and inhaled, with a worsening of the ischemic damage [19]. Another factor that may have a negative impact on the possible success of an endovascular procedure in patients with ischemic stroke could include the need for conversion from conscious sedation to general anesthesia during the procedure. Simonsen et al. focused on this point, proving a worse general outcome in patients needing conversion. Nevertheless, their meta-analysis failed to identify possible factors related to an increased risk of conversion [14]. A similar conclusion emerged from a trial conducted by Chen et al., with, in addition, the demonstration of a reduction in the success rate of the revascularization procedure in patients needing a conversion to general anesthesia and an emergency endotracheal intubation [20]. On the contrary, Flottmann et al. in their trial proved no differences in patients needing an emergency conversion to general anesthesia in terms of the survival rate and success of the revascularization procedure, compared to those treated with general anesthesia from the beginning of the procedure [21]. Brinjikji et al. also observed no impact of anesthesiologic conduct on the time required for the revascularization procedure, besides the fundamental advantage of neurological monitoring during the procedure allowed by conscious sedation [22]. Some authors deepened the modality of choice of the anesthesiological conduct. An interesting analysis regarding the situation in various centers in Spain was performed by Kräuchi et al. In particular, in the majority of cases, the choice of anesthesia was shared with neuroradiologists, in accordance with the clinical conditions of patients, such as cardiovascular or respiratory parameters. The same theory of the individualization of anesthesia strategy is shared by Talke et al. in their study [23,24].

## 5. Conclusions

The rate of success of revascularization procedures is almost overlapping between patients treated with conscious sedation and general anesthesia. In addition, we did not notice significant differences between groups in terms of functional and clinical outcomes, using various scores as references, such as the National Institutes of Health Stroke Scale (NIHSS) score at 7 days, NIHSS and Modified Rankin Scale (MRS) at time of discharge, and MRS after 3 months. Considering the possible usefulness of applying conscious sedation, at OCSAE of Baggiovara, an internal protocol for conscious sedation was introduced to standardize the treatment in patients undergoing endovascular procedures.

## 6. Strengths and Limitations

It is important to note that this study has limitations due to its retrospective nature, its small sample size, and it being conducted at a single center. However, the findings from our study align with those expressed in other works and provide valuable insights into the topic.

## 7. Ethics

All patients enrolled in this study, after having been admitted to our center for ischemic stroke to be submitted for endovascular therapy, provided specific written informed consent.

## Figures and Tables

**Table 1:** Population of this study. The table reports the main comorbidities of the population of our study and the ischemic lesion site in the 3 groups. Multiple comorbidities may be present in the same patient, which is why the total percentage may be greater than 100%. The mean (±SD) is reported. The following abbreviations are used: AG for general anesthesia, SC for conscious sedation, AL for local anesthesia, ACA for anterior cerebral artery, MCA for middle cerebral artery, PCA for posterior cerebral artery, VA for vertebral artery, BA for basilar artery, ICA for internal carotid artery.

Hospital Admission	Overall (58 Patients)	General Anesthesia (25.86%)	Conscious Sedation (46.55%)	Local Anesthesia (27.59%)
Comorbidities				
Cardiovascular	53 (91.23%)	13 (92.86%)	25 (92.59%)	14 (87.50%)
Respiratory	13 (22.81%)	5 (35.71%)	5 (18.52%)	3 (18.75%)
Kidney failure	4 (7.02%)	0 (0%)	2 (7.41%)	2 (12.50%)
Gastrointestinal	15 (26.32%)	3 (21.43%)	6 (22.6%)	6 (37.50%)
Neoplastic	5 (8.77%)	1 (7.14%)	3 (11.11%)	1 (6.25%)
Neurologic	16 (28.07%)	3 (21.43%)	9 (33.3%)	4 (25%)
Metabolic	8 (14.04%)	1 (7.14%)	5 (18.52%)	2 (12.50%)
Ischemic lesion site				
ACA	1 (1.72%)	0 (0)	1 (3.70%)	0 (0)
MCA	36 (62.07%)	6 (40%)	18 (66.67%)	12 (75%)
PCA, VA, BA	7 (12.07%)	6 (40%)	0 (0)	1 (6.25%)
ICA	5 (8.62%)	1 (6.67%)	3 (11.11%)	2 (12.50%)
ICA, MCA	7 (12.07%)	2 (13.33%)	3 (11.11%)	2 (12.50%)
MCA, ACA	2 (3.45%)	0 (0)	2 (7.41%)	0 (0)

**Table 2:** Variables at admission. GA refers to general anesthesia, CS refers to conscious sedation, and LA refers to local anesthesia. The following admission parameters are measured: GCS for Glasgow Coma Scale score, NIHSS for National Institutes of Health Stroke Scale, MRS for Modified Rankin Scale, MEWS for Modified Early Warning Score, RASS for Richmond Agitation and Sedation Scale, SOFA SCORE for Sequential Organ Failure Assessment Score.

	Overall (58 Patients)	General Anesthesia (25.86%)	Conscious Sedation (46.55%)	Local Anesthesia (27.59%)	*p* Value
Age	71.22 ± 17.11	65.43 ± 17.45	74.89 ± 14.9	70.47 ± 20.65	0.227
GCS Admission	12.30 ± 2.36	12.15 ± 3.38	12.29 ± 1.91	12.43 ± 2.20	0.9513
NIHSS Admission	14.91 ± 6.74	15.50 ± 7.81	14.74 ± 6.15	14.68 ± 7.11	0.939
MRS Admission	0.21 ± 0.52	0.00 ± 0	0.40 ± 0.69	0.06 ± 0.25	0.023
MEWS Admission	1.40 ± 1.5	1.28 ± 1.13	1.44 ± 1.55	1.43 ± 1.86	0.948
RASS	0.00 ± 0.73	−0.14 ± 1.23	0.07 ± 0.61	0.00 ± 0.0	0.675
SOFA Admission	2.26 ± 1.75	2.00 ± 1.75	2.59 ± 1.64	1.93 ± 1.98	0.4116

**Table 3:** Outcomes of this study. The following abbreviations are used: AG for general anesthesia, SC for conscious sedation, AL for local anesthesia, GCS for Glasgow Coma Scale, NIHSS for National Institutes of Health Stroke Scale, MAP for Mean Arterial Pressure, SOFA SCORE for Sequential Organ Failure Assessment Score.

	Overall (58 Patients)	General Anesthesia (25.86%)	Conscious Sedation (46.55%)	Local Anesthesia (27.59%)	*p* Value
Lactates after the procedure	1.99 ± 3.06	2.23 ± 3.88	1.68 ± 1.01	1.35 ± 0.35	0.902
GCS before the procedure	12.16 ± 2.51	12.25 ± 2.70	11.81 ± 2.60	12.68 ± 2.27	0.549
NIHSS before the procedure	14.51 ± 6.63	16.55 ± 7.012	13.55 ± 6.12	14.56 ± 7.29	0.428
MAP before the procedure	102.33 ± 11.97	103.64 ± 12.69	100.53 ± 11.82	104.38 ± 11.89	0.411
MAP during the procedure	102.33 ± 11.97	96.09 ± 19.05	95.8 ± 15.76	99.52 ± 12.59	0.582
MAP after the procedure	89.44 ± 14.11	86.50 ± 16.58	90.01 ± 14.70	91.06 ± 10.99	0.657
GCS after the procedure	12.58 ± 2.6	11.50 ± 3.66	12.76 ± 2.26	12.81 ± 3.09	0.492
SOFA after the procedure	2.68 ± 2.13	3.42 ± 2.20	2.40 ± 1.81	2.50 ± 2.52	0.325
NIHSS after 7 days	10.04 ± 8.71	7.85 ± 4.59	11.62 ± 9.05	8.2 ± 8.53	0.374
NIHSS at discharge	7.61 ± 9.03	9.80 ± 9.16	8.38 ± 9.56	4.38 ± 7.48	0.301
MRS at discharge	3.26 ± 2.00	4.08 ± 1.72	3.19 ± 1.78	2.73 ± 2.43	0.215
MRS after 3 months	3.20 ± 2.25	4.75 ± 2.50	2.06 ± 2.02	3.5 ± 2.36	0.215
Duration of the procedure	95.59 ± 71.95	144.44 ± 94.97	86.16 ± 59.84	80.35 ± 66.31	0.072
Survival rate	52 (84.48%)	13 (25%)	25 (48.8%)	14 (26.92%)	0.824

**Figure A1:**
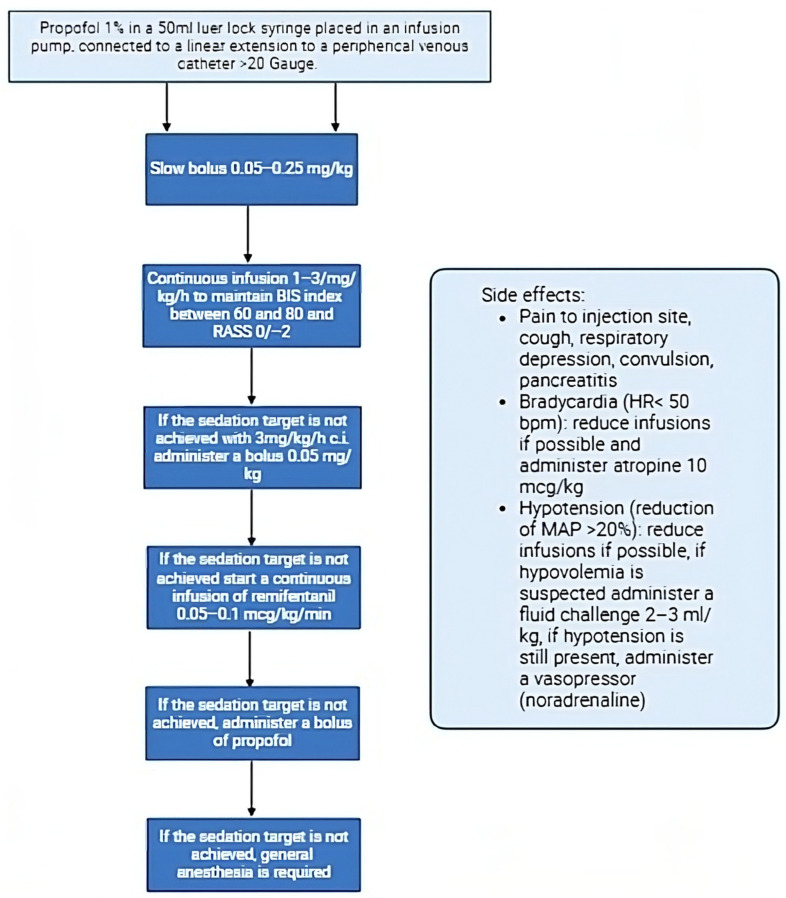
Protocol suggested by l’AOU OCSAE di Baggiovara (MO) equipe for conscious sedation.

## Data Availability

Dataset available on request from the authors.

## Appendix A

In Figure A1, we show the protocol suggested by l’AOU OCSAE di Baggiovara (MO) equipe for conscious sedation for mechanical trombectomy in patients with acute stroke.
